# The interplay of UBE2T and Mule in regulating Wnt/β-catenin activation to promote hepatocellular carcinoma progression

**DOI:** 10.1038/s41419-021-03403-6

**Published:** 2021-02-01

**Authors:** Nicole Pui Yu Ho, Carmen Oi Ning Leung, Tin Lok Wong, Eunice Yuen Ting Lau, Martina Mang Leng Lei, Etienne Ho Kit Mok, Hoi Wing Leung, Man Tong, Irene Oi Lin Ng, Jing Ping Yun, Stephanie Ma, Terence Kin Wah Lee

**Affiliations:** 1 Department of Applied Biology and Chemical Technology, The Hong Kong Polytechnic University, Hong Kong, Hong Kong; 2 School of Biomedical Sciences, Li Ka Shing Faculty of Medicine, The University of Hong Kong, Hong Kong, Hong Kong; 3 Department of Clinical Oncology, Queen Elizabeth Hospital, Hong Kong, Hong Kong; 4 Department of Pathology, Queen Mary Hospital, The University of Hong Kong, Hong Kong, Hong Kong; 5 State Key Laboratory of Liver Research, The University of Hong Kong, Hong Kong, Hong Kong; 6 Department of Pathology, Sun Yat Sen University Cancer Center, Guangzhou, China; 7 State Key Laboratory of Chemical Biology and Drug Discovery, The Hong Kong Polytechnic University, Hong Kong, Hong Kong

**Keywords:** Cancer stem cells, Liver cancer

## Abstract

Emerging evidence indicates the role of cancer stem cells (CSCs) in tumor relapse and therapeutic resistance in patients with hepatocellular carcinoma (HCC). To identify novel targets against liver CSCs, an integrative analysis of publicly available datasets involving HCC clinical and stemness-related data was employed to select genes that play crucial roles in HCC via regulation of liver CSCs. We revealed an enrichment of an interstrand cross-link repair pathway, in which ubiquitin-conjugating enzyme E2 T (UBE2T) was the most significantly upregulated. Consistently, our data showed that UBE2T was upregulated in enriched liver CSC populations. Clinically, UBE2T overexpression in HCC was further confirmed at mRNA and protein levels and was correlated with advanced tumor stage and poor patient survival. UBE2T was found to be critically involved in the regulation of liver CSCs, as evidenced by increases in self-renewal, drug resistance, tumorigenicity, and metastasis abilities. Mule, an E3 ubiquitin ligase, was identified to be the direct protein binding partner of UBE2T. Rather than the canonical role of acting as a mediator to transfer ubiquitin to E3 ligases, UBE2T is surprisingly able to physically bind and regulate the protein expression of Mule via ubiquitination. Mule was found to directly degrade β-catenin protein, and UBE2T was found to mediate liver CSC functions through direct regulation of Mule-mediated β-catenin degradation; this effect was abolished when the E2 activity of UBE2T was impaired. In conclusion, we revealed a novel UBE2T/Mule/β-catenin signaling cascade that is involved in the regulation of liver CSCs, which provides an attractive potential therapeutic target for HCC.

## Introduction

Liver cancer (hepatocellular carcinoma, HCC) is one of the deadliest diseases and is the fourth leading cause of cancer-related mortality worldwide^1^. Treatment modalities of HCC commonly include surgical resection, transarterial chemoembolization, chemotherapy, and molecularly targeted drugs^2^. However, the survival benefits are modest, attributed to frequent tumor recurrence and the development of chemoresistance. Compelling evidence has emerged in support of the vast contribution of cancer stem cells (CSCs; also known as tumor-initiating cells, T-ICs) to the poor survival of cancer patients as they promote chemoresistance, tumorigenicity, and metastasis^3–7^. Therefore, it is critical to identify new therapeutic targets against CSCs for the benefit of HCC patients. Integrative comparative genomic analysis has established molecular similarities between liver CSCs and normal liver stem cells^8^, suggesting the importance of liver CSCs in hepatocarcinogenesis. Identification of critical molecules/pathways involved in the regulation of liver CSCs may prolong the survival of HCC patients. For this purpose, we have employed an integrative analysis of publicly available datasets involving HCC clinical and stemness-related data to select genes that play crucial roles in HCC via the regulation of liver CSCs. This analysis showed that ubiquitin-conjugating enzyme E2 T (UBE2T), among other genes in the interstrand cross-link repair pathway, was drastically upregulated and activated in self-renewing HCC cells compared to control cells.

UBE2T is the E2 ubiquitin-conjugating enzyme of the (Fanconi anemia) FA DNA repair pathway, which binds with different E3 ligases to promote the ubiquitination of target proteins^9^. UBE2T was first identified in CD34^+^ hematopoietic stem cells, suggesting its regulatory role in the stemness properties of these cells^10^. More recently, UBE2T was found to regulate the progression of stomach and nasopharyngeal cancers at least in part through modulation of the Wnt signaling cascade^11,12^. Although these findings suggest a potential role of UBE2T in regulating stemness, the molecular mechanism by which UBE2T regulates stemness properties is poorly understood. Whether UBE2T regulates liver CSCs also remains unexplored. In this study, we found that UBE2T plays a crucial role in the regulation of liver CSC functions. First, we found that overexpression of UBE2T at both the mRNA and protein levels was associated with advanced tumor stages and poor patient survival. UBE2T was found to regulate the phenotypes of liver CSCs, including self-renewal, drug resistance, tumorigenicity, and metastasis. By tandem affinity purification coupled with mass spectrometry, we identified Mcl-1 ubiquitin ligase E3 (Mule) as the direct binding partner of UBE2T, which regulates ubiquitination of β-catenin. Collectively, our study identified a novel noncanonical Wnt/β-catenin signaling cascade mediated by the UBE2T/Mule complex. Targeting the UBE2T/Mule/β-catenin signaling cascade is a new potential therapeutic approach for HCC.

## Materials and methods

### Cell lines and cell culture

Human HCC cell lines MHCC-97L (Liver Cancer Institute, Fudan University), Hep3B (ATCC), Huh7 and PLC/PRF/5 (Japan Cancer Research Bank), MIHA (a gift from Dr. J.R. Chowdhury, Albert Einstein College of Medicine) and HEK293T (ATTC) were maintained in Dulbecco’s Modified Eagle Medium (DMEM) with high glucose and l-glutamine (Gibco, Invitrogen) supplemented with 10% heat-inactivated fetal bovine serum (Gibco, Invitrogen), 100 mg/mL penicillin G, and 50 µg/mL streptomycin (Gibco, Invitrogen) at 37 °C in a humidified chamber containing 5% CO_2_. Lentiviral infected cells were cultured in complete DMEM medium supplemented with 1 µg/mL puromycin. Culture medium was refreshed every 2 days. All cell lines used in this study were obtained between 2013 and 2016, regularly authenticated by morphologic observation and AuthentiFiler STR (Invitrogen) and tested for absence of mycoplasma contamination (MycoAlert, Lonza). Cells were used within 20 passages after thawing.

### Human tissue specimens for mRNA expression analysis

Paired patient HCC and adjacent noncancerous liver tissue specimens were collected at the time of surgical resection from patients at Queen Mary Hospital, Hong Kong, from 1991 to 2013. The use of human clinical specimens was approved by the Institutional Review Board of the University of Hong Kong/Hospital Authority Hong Kong West Cluster. Consent from patients was obtained. Sample size of human HCC samples was chosen based on G-power calculation.

### Tandem affinity purification coupled with mass spectrometry

Stable clones of HEK293T cells expressing UBE2T with N-terminally tagged with SFB were harvested and lysed in NETN buffer for 30 mins on ice. After removal of cell debris by centrifugation at 14K rpm for 15 mins, supernatant containing UBE2T-protein complexes was first immunoprecipitated by streptavidin bead slurry (Amersham) for 4 h at 4 °C. The precipitated protein complexes were then eluted by incubation with NETN buffer containing 2 mg/ml biotin (Sigma-Aldrich). The biotin-eluate was then incubated with S protein agarose (Novagen) for 3 h to perform a second round of immunoprecipitation. The protein complexes were eluted by heating at 95 °C for 5 mins. The eluate was subjected to mass spectrometric analysis (LC/MS/MS) at Taplin Mass Spectrometry Facility (Harvard).

### Metastatic In vivo orthotropic implantation

A total of 1 × 10^6^ luciferase-labeled MHCC-97L cells (NTC vs shUBE2T) resuspended in matrigel (DMEM: matrigel 50:50) were injected into the left lobes of livers of 5–7-week-old male BALB/C nude mice. Six weeks after implantation, mice were administered with 100 mg/kg D-luciferin via peritoneal injection 5 mins before bioluminescent imaging (IVIS Lumina Series III Pre-clinical In Vivo Animal Imaging System, Perkin-Elmer). Lungs and livers were harvested for ex vivo imaging. The study protocol was approved by and performed in accordance with the guidelines for the Use of Live Animals in Teaching and Research at Hong Kong Polytechnic University. No specific randomization method was used. Sample size of animals was chosen based on significant *p* values.

### Tumorigenicity assay

In vivo evaluation of tumorigenicity was performed with 5–7-week-old NOD-SCID mice by induction of tumor xenografts. Cells were suspended in 1:1 culture medium and BD Matrigel Matrix (BD Biosciences) and subcutaneously injected into the flanks of the NOD-SCID mice, which were kept under observation. Briefly, each mouse received two injections of cells in both flanks, and cells from each experimental group (NTC vs shUBE2T; EV vs UBE2T OE) were injected into different mice. Tumors were harvested at the end of the experiment for documentation. Tumor-initiating cell frequency was calculated using Extreme Limiting Dilution Analysis (ELDA) software. The study protocol was approved by and performed in accordance with the guidelines for the Use of Live Animals in Teaching and Research at Hong Kong Polytechnic University. No specific randomization method was used. Sample size of animals was chosen based on significant *p* values.

### Statistical analysis

Statistical significance of qPCR, sphere formation assay, flow cytometry analysis, migration assay and invasion assay results were determined by Student’s *t* test using Microsoft Office Excel software (Microsoft Corporation). The displayed results showed the means and the standard deviations, and those with *p* values less than 0.05 were considered statistically significant (*p < 0.05, **p < 0.01, ***p < 0.001). All tests are two-sided. Data point is excluded if it deviates from mean with more than three standard deviations. Investigators were not blinded to the group allocation during experiment and when assessing the outcome in all experiments including animal experiments. There is no estimate of variation within each group of data. Variance is similar between the groups that are being statistically compared. Chi-square test was used to evaluate correlations between clinico-pathological parameters and UBE2T expression. Kaplan–Meier survival analysis was used to analyze overall survival and disease-free survival and a log-rank test was used to determine the statistical significance; these analyses were carried out using SPSS 20 software.

Additional experimental procedures are provided in the Supplementary Methods.

## Results

### UBE2T is overexpressed in enriched liver CSC populations and human HCC specimens

**Fig. 1:**
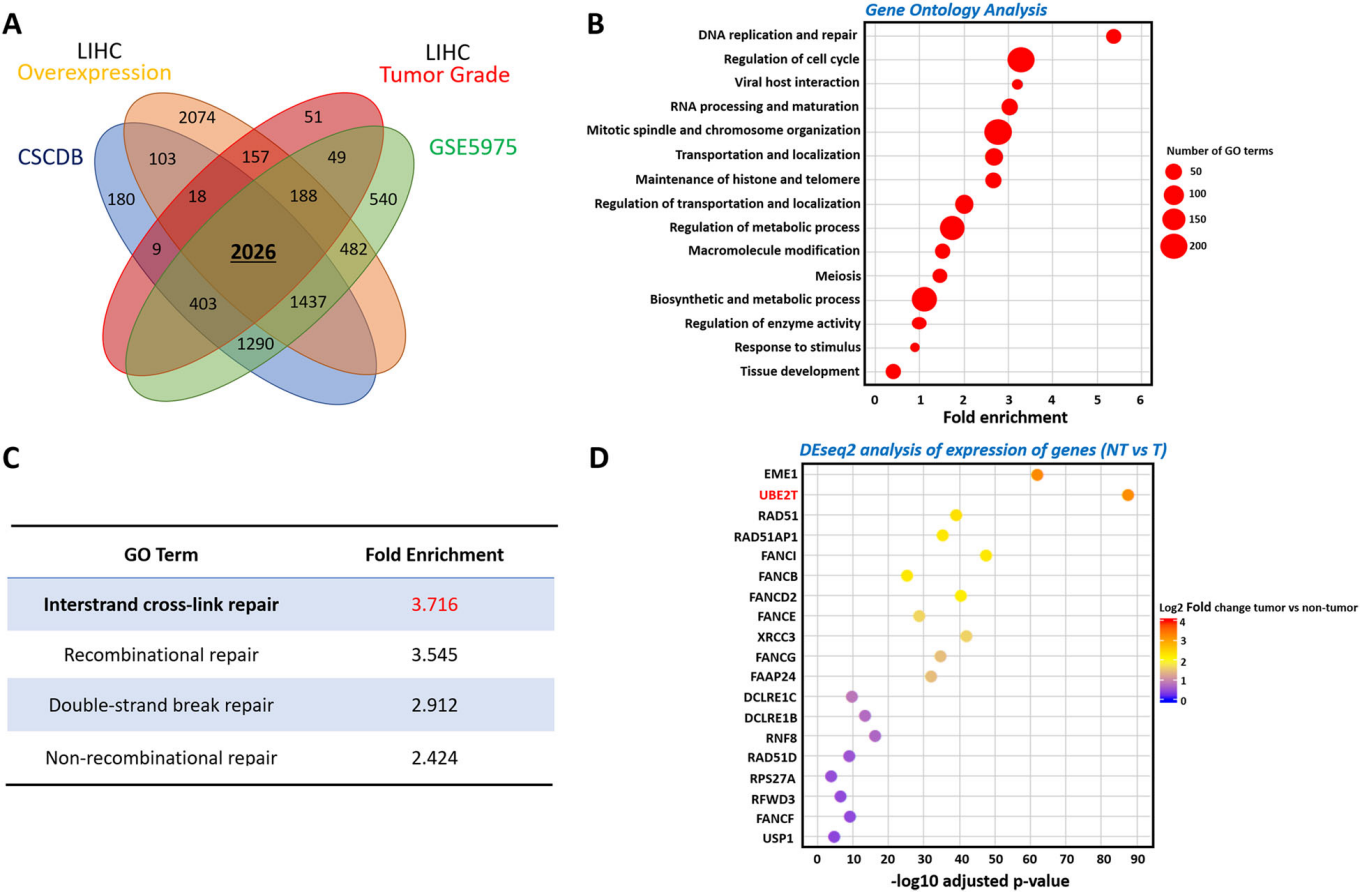
UBE2T was upregulated in enriched liver CSC populations. **a** An integrative analysis of three publicly available datasets, CSCdB, TCGA (LIHC), and GSE5975, was performed to search for genes that were commonly upregulated between liver cancer cells and liver CSCs compared to normal liver cells. A Venn diagram analysis showed that a total of 2026 genes were commonly upregulated. **b** GO analysis of these 2026 genes with BiNGO followed by clustering with AutoAnnotate revealed enrichment of DNA replication and repair pathways. **c** Within the replication and repair pathway, the interstrand cross-link repair pathway had the highest and most significant fold enrichment. **d** Upon DEseq2 analysis, UBE2T was the most significantly upregulated gene among 19 genes in the interstrand cross-link repair pathway.

**Fig. 2:**
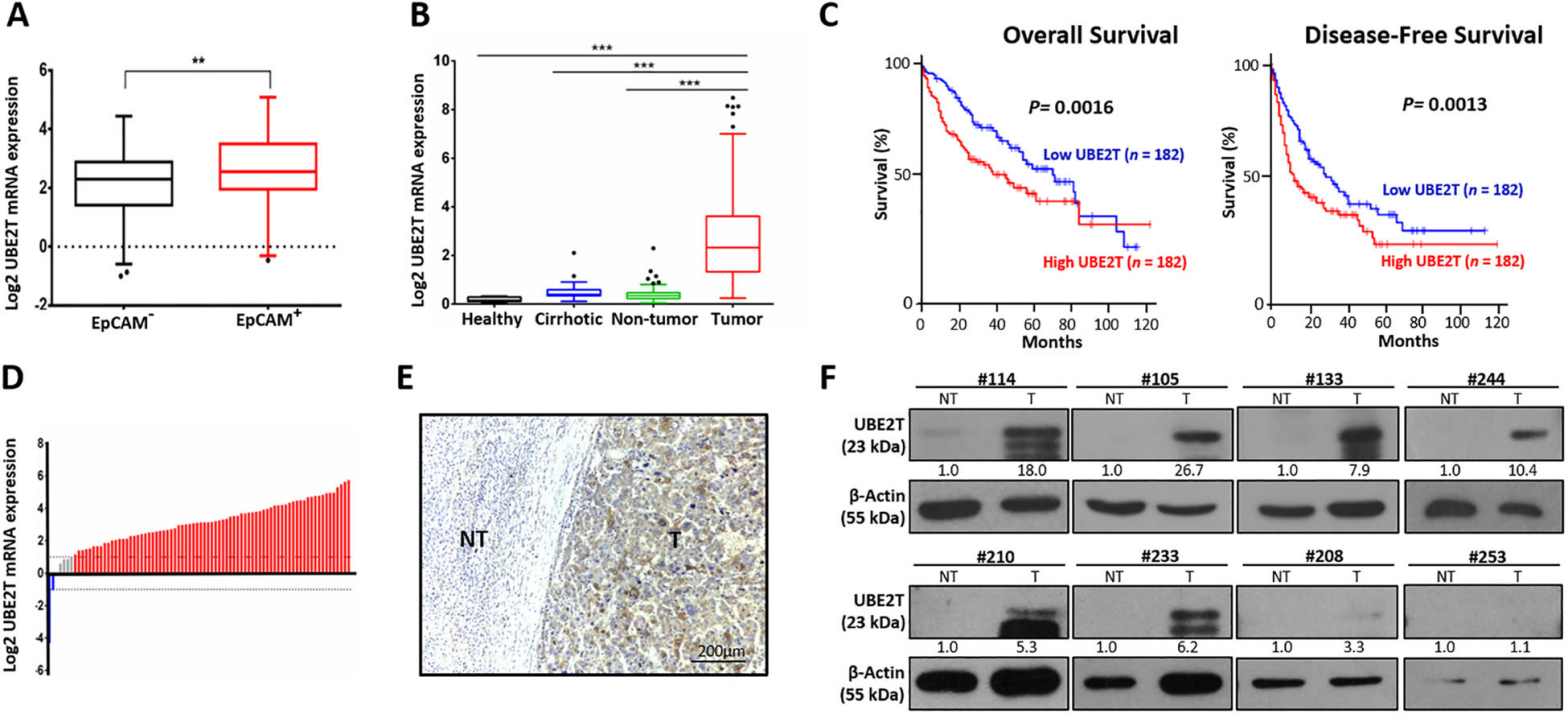
UBE2T is upregulated in the enriched CSC population and contributes to poor patient survival. **a** UBE2T expression was significantly upregulated in CSC-enriched EpCAM^+^ populations in the GSE5975 dataset (**p < 0.01, *t* test). **b** In the analysis of dataset GSE25097, we found upregulation of UBE2T mRNA in a cohort of 243 paired HCC and nontumor samples (***p < 0.001, *t* test). In addition, UBE2T was upregulated in cirrhotic samples compared with healthy donor samples (*p < 0.05, *t* test). **c** In the analysis of the Cancer Genome Atlas (TCGA), the overall and disease-free survival rates of HCC patients with high UBE2T overexpression were significantly lower than those of patients with low UBE2T expression (*p* = 0.0016 and *p* = 0.0013, respectively; log-rank test). **d** qPCR analysis showed that 55% of HCC patient specimens (45 out of 82 cases) showed an eightfold increase in UBE2T mRNA expression compared to that in adjacent nontumor liver tissues. **e** A representative photo (case #12457) showing the boundary between the nontumor (NT) and tumor (T) regions (M indicates the margin, scale bar: 200 µm). **f** UBE2T protein overexpression was found in 87.5% (7/8) of HCC specimens (*n* = 8). Immunoblots are representative of two individual experiments.

To screen for critical molecules/pathways involved in the regulation of liver CSCs, we first analyzed the genes that are upregulated in enriched liver CSC populations and HCC clinical samples compared to normal control samples. For this purpose, we performed an integrative analysis of three publicly available datasets involving HCC clinical (liver hepatocellular carcinoma (the Cancer Genome Atlas (TCGA)-LIHC) data and stemness-related data (GSE5975 and CSCdb))^13,14^. First, we searched for genes that were upregulated in HCC that were related to liver CSC signatures and poor differentiation. By this means, we were able to identify 2026 genes that were commonly upregulated (Fig. 1a, Supplementary Table S1). Gene ontology analysis with BiNGO followed by clustering with AutoAnnotate showed that DNA replication and repair were the highest ranked pathways (Fig. 1b), among which interstrand cross-link repair showed the greatest enrichment (Fig. 1c). Out of the 19 genes related to interstrand cross-link repair, UBE2T was the most significantly upregulated gene (Fig. 1d). In parallel with this finding, we found that UBE2T expression was upregulated in enriched liver CSC populations marked by EpCAM in the publicly available dataset (GSE5975) consisting of 238 HBV-positive HCC clinical specimens, with normal specimens as the control (Fig. 2a). In the analysis of another dataset (GSE25097), UBE2T was significantly upregulated in tumor specimens from normal to cirrhotic to tumor tissues in a stepwise manner, suggesting an oncogenic role for UBE2T in liver carcinogenesis (Fig. 2b). In addition, TCGA data analysis of 442 HCC patients showed that patients with high UBE2T expression (above the median level) had poorer overall survival (*p* = 0.0016, log-rank test) and disease-free survival (*p* = 0.0013, log-rank test) than patients with low UBE2T expression (Fig. 2c). To further validate the results, we evaluated UBE2T mRNA expression in a cohort of 82 patients via qPCR analysis. 91% (75/82) of the patients showed more than twofold upregulation of UBE2T mRNA levels, while 55% (45/82) of the patients showed more than eightfold upregulation in HCC samples compared to normal samples (Fig. 2d). Statistical analysis by SPSS software revealed that higher expression of UBE2T was associated with larger tumor size (*p* = 0.023), venous invasion (*p* = 0.049), and advanced tumor stage (*p* = 0.045) (Supplementary Table S2). Consistently, UBE2T was found to be overexpressed at the protein level by IHC staining and Western blot analysis (Fig. 2e, f). Collectively, these results provide solid evidence that UBE2T is upregulated in liver CSCs and has clinical significance in HCC.

### UBE2T regulates self-renewal, tumorigenicity, and liver CSC marker expression in HCC cells

**Fig. 3:**
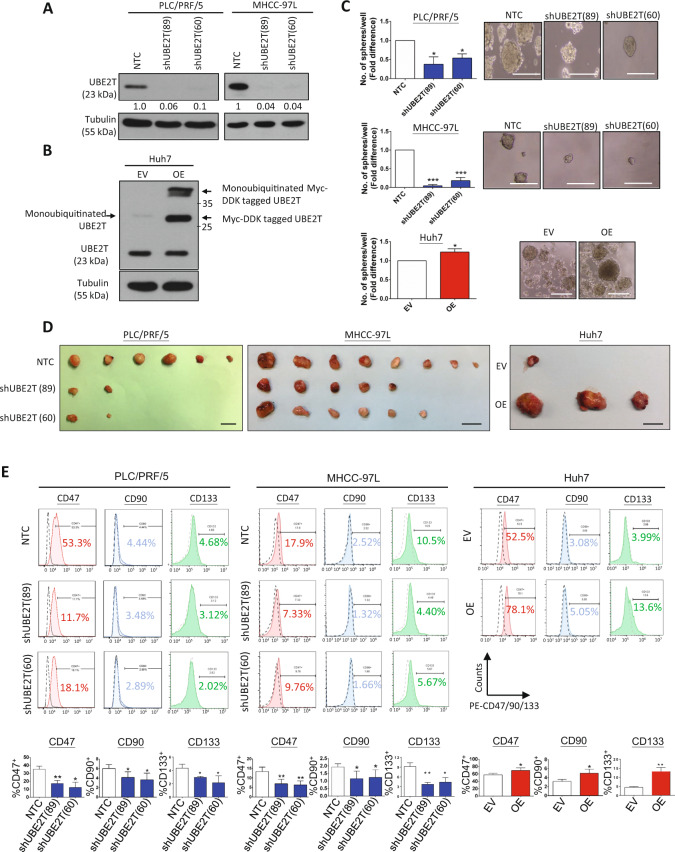
UBE2T regulates the liver CSC characteristics of HCC cells. **a** Two different shUBE2T sequences (89 and 60) and one sequence for nontarget control (NTC) were used. Western blotting showed the successful knockdown of UBE2T in PLC/PRF/5 and MHCC-97L cells. **b** Successful overexpression of UBE2T was also evidenced in Huh7 cells. **c** Knockdown of UBE2T also reduced the size and number of hepatospheres formed by PLC/PRF/5 and MHCC-97L cells (*p < 0.05 and ***p < 0.001, *t* test), while overexpression of UBE2T increased the size and number of hepatospheres formed by Huh7 cells (*p < 0.05, *t* test; scale bar: 100 µm). **d** Knockdown of UBE2T in PLC/PRF/5 and MHCC-97L cell lines suppressed tumorigenicity compared with that in NTC cells. Representative photos showing the injection of 50000 and 10000 cells derived from PLC/PRF/5 and MHCC-97L cells. Overexpression of UBE2T led to increased tumorigenicity of Huh7 cells. Representative photographs showing the injection of 10000 cells (scale bar: 1 cm). **e** Knockdown of UBE2T decreased the expression of CD47, CD90, and CD133 in both PLC/PRF/5 and MHCC-97L cells compared with that in NTC cells (*p < 0.05 and **p < 0.01, *t* test). Overexpression of UBE2T increased the expression of CD47, CD90, and CD133 in Huh7 cells (*p < 0.05 and **p < 0.01, *t* test). Immunoblots, FACS and sphere formation assay represent means ± SD in at least three independent experiments (*n* = 3–4).

To further examine whether UBE2T functionally contributes to the traits of liver CSCs, we performed UBE2T-knockdown and UBE2T-overexpression experiments using a lentivirus-based approach. By Western blotting, we found high expression of UBE2T in a panel of HCC cell lines, including MHCC-97L and PLC/PRF/5 (Supplementary Fig. S1); therefore, these two cell lines were chosen for the knockdown experiment, while Huh7 cells were chosen for the overexpression experiment due to relatively low UBE2T expression. Western blotting was used to confirm the successful establishment of the knockdown and overexpression clones (OE) (Fig. 3a, b). Knockdown of UBE2T reduced the spheroid-forming ability of both PLC/PRF/5 and MHCC-97L cells by an average of 55% and 88%, respectively, while UBE2T overexpression was shown to enhance the spheroid-forming ability of Huh7 cells by an average of 23% (Fig. 3c). Next, we evaluated the role of UBE2T in liver CSCs in vivo. Limiting dilution analysis was performed to determine how UBE2T affects the tumorigenicity of HCC cells and the CSC frequency. In both PLC/PRF/5 and MHCC-97L cells, knockdown of UBE2T reduced the size and number of tumors formed (Fig. 3d, Supplementary Table S3). Conversely, UBE2T overexpression in Huh7 cells significantly enhanced tumorigenicity, with an increase in the estimated CSC frequency (Fig. 3d, Supplementary Table S3). By flow cytometry analysis, the expression of two liver CSC markers, CD47, CD133 and CD90, on HCC cells was examined upon UBE2T knockdown and overexpression. The suppression of UBE2T reduced the percentages of CD47-, CD133- and CD90-positive PLC/PRF/5 and MHCC-97L cells, while UBE2T-overexpressing Huh7 cells exhibited increases in the percentages of these markers (Fig. 3e).

### UBE2T augments the chemoresistance and metastatic potential of HCC cells

**Fig. 4:**
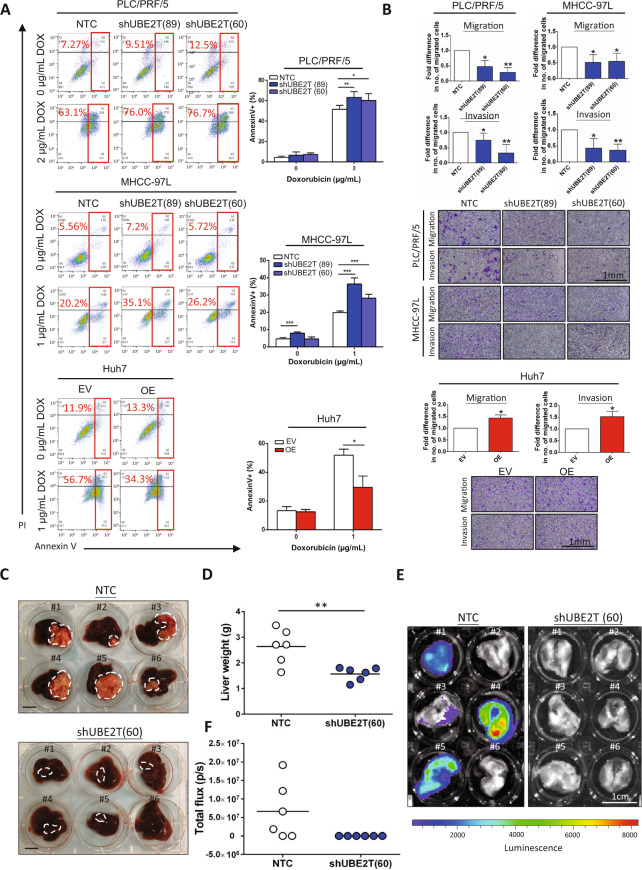
UBE2T regulates the chemoresistance, migration, invasion, and metastatic potential of HCC cells. **a** Compared to NTC cells, shUBE2T cells derived from PLC/PRF/5 and MHCC-97L cells showed a higher percentage of annexin V-positive cells in response to doxorubicin treatment at 2 and 1 µg/mL for 24 h. Compared to EV control cells, UBE2T OE cells showed a lower percentage of annexin V-positive cells in response to doxorubicin treatment at 1 µg/mL for 24 h (*p < 0.05, **p < 0.01, and ***p < 0.001, *t* test). **b** Knockdown of UBE2T reduced the number of migratory and invasive HCC cells in uncoated and Matrigel-coated Transwell assays, respectively (*p < 0.05 and **p < 0.01, *t* test). UBE2T OE cells showed a higher number of migratory and invasive HCC cells than EV control cells (*p < 0.05, *t* test). **c** In an orthotopic HCC metastatic model, livers with corresponding tumors derived from NTC cells and shUBE2T (60) cells were excised (scale bar: 1 cm). **d** Liver weight was shown as a dot plot (**p < 0.05, *t* test). **e** Suppression of UBE2T reduced the incidence of lung metastasis (0/6 vs. 4/6) in MHCC-97L cells in vivo. **f** Signal intensity of the lungs was shown as a dot plot. Apoptotic assay, migration and invasion assays represent means ± SD in at least three independent experiments (*n* = 3–6).

Next, we explored the effect of UBE2T alteration on the chemoresistance of HCC cells. We evaluated the sensitivity of HCC cells to doxorubicin treatment using an annexin V assay. Knockdown of UBE2T enhanced the cell death of HCC cells upon doxorubicin treatment (Fig. 4a). Similar findings were observed in shUBE2T cells in response to sorafenib and lenvatinib treatments (Supplementary Fig. S2). Next, we investigated whether the role of UBE2T in sorafenib resistance is clinically relevant by analyzing 30 HCC patients who received prior sorafenib treatment in TCGA-LIHC dataset. Consistently, patients with high UBE2T expression were correlated with shorter overall survival (*p* = 0.0299, log-rank test), which is independent of tumor staging (Supplementary Fig. S3). In contrast, UBE2T-overexpressing Huh7 cells exhibited reduced cell death when treated with doxorubicin (Fig. 4a). In Supplementary Table S2, UBE2T overexpression was correlated with venous invasion. Consistently, transwell assays showed that UBE2T knockdown decreased the migration and invasion abilities of PLC/PRF/5 and MHCC-97L cells, while UBE2T overexpression exerted the opposite effects (Fig. 4b). To further confirm whether UBE2T also promotes metastatic potential in vivo, an orthotopic HCC xenograft model was employed in which luciferase-labeled MHCC-97L cells derived from shUBE2T and corresponding control cells were directly implanted into the left lobe of the liver. When compared with control tumors, we found that UBE2T-repressed MHCC-97L cells exhibited a drastic reduction in tumor volume, as evidenced by an increase in liver weight (Fig. 4c, d). In addition, four out of six mice (~67%) showed lung metastasis in this orthotopic HCC xenograft model, while none of the mice injected with shUBE2T MHCC-97L cells showed lung metastasis (Fig. 4e, f).

### UBE2T physically binds to Mule and regulates its protein expression via ubiquitination

**Fig. 5:**
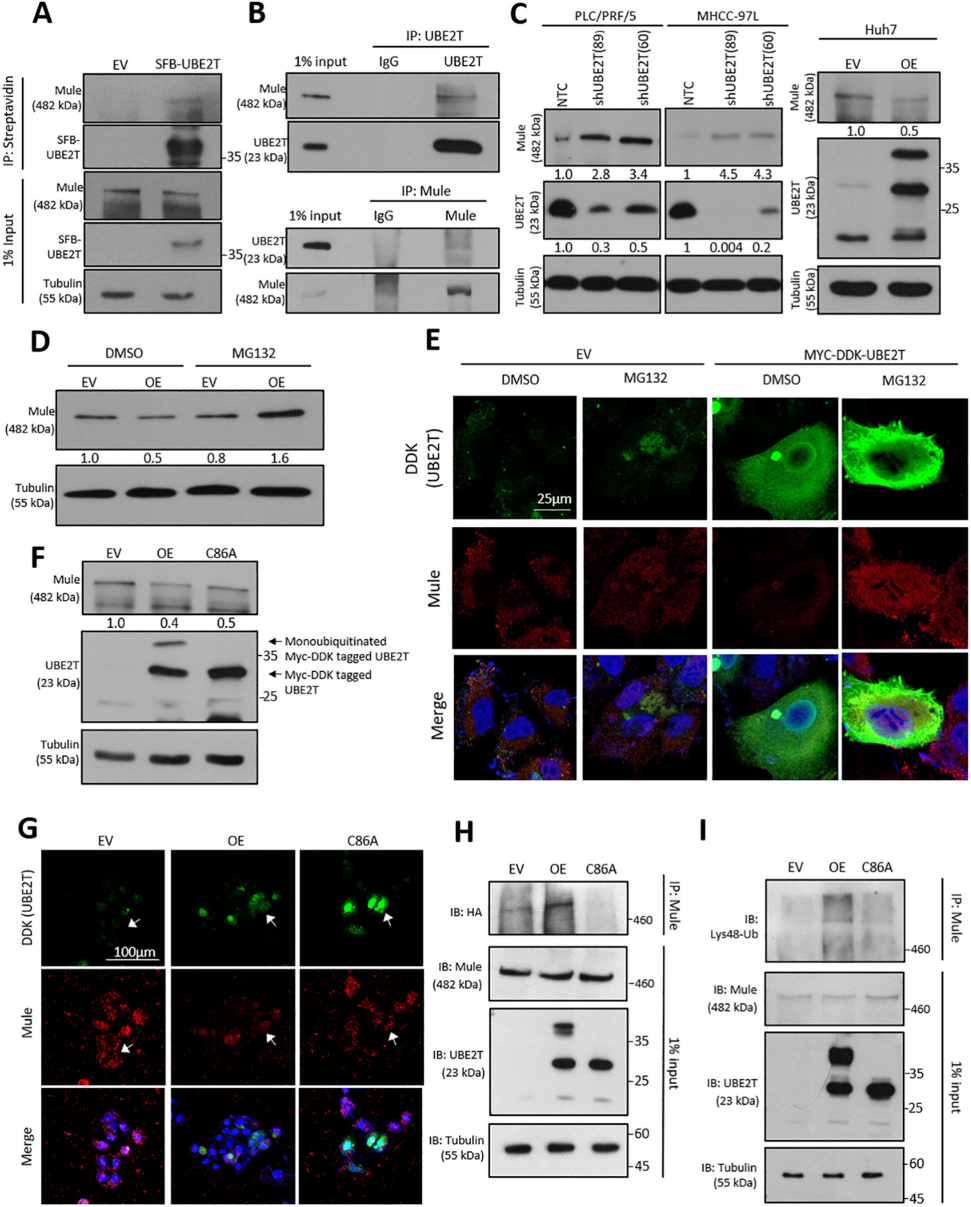
Functional significance of the interaction of UBE2T with Mule. **a** The interaction between SFB-UBE2T and Mule was demonstrated by coimmunoprecipitation in Huh7 cells. Mule was detected after the pull-down of SFB-UBE2T. **b** Reciprocal coimmunoprecipitation demonstrated the interaction between endogenous UBE2T and Mule in MHCC-97L cells. **c** Mule protein levels were upregulated upon UBE2T knockdown in PLC/PRF/5 and MHCC-97L cells. UBE2T overexpression led to the downregulation of Mule in Huh7 cells. **d** UBE2T-overexpressing Huh7 cells were subjected to MG132 treatment. Western blot analysis showed that the proteasome inhibitor MG132 rescued the downregulation of Mule in UBE2T-overexpressing cells. **e** IF analysis showed a reduction in Mule expression in UBE2T OE cells compared to control cells. MG132 treatment increased the level of Mule in both the cytoplasm and nucleus, indicating that UBE2T regulates Mule by proteasomal degradation (scale bar: 25 μm). **f** Impaired E2 activity of UBE2T reduced the degradation of Mule. The arrow indicates the absence of monoubiquitination in the C86A mutant. **g** By IF staining, the C86A mutation rescued the suppression of Mule expression in the UBE2T OE clones of Huh7 cells (scale bar: 100 μm). **h** HA-ubiquitin together with wild-type Myc-DDK-UBE2T, C86A mutant or control vector were co-transfected into Huh7 cells. After treatment with 20 μM MG132 for 6 h, Mule was pulled down to investigate the effect of UBE2T on the abundance of ubiquitinated Mule. Overexpression of wild-type UBE2T promoted the ubiquitination of Mule, while C86A mutation significantly suppressed ubiquitination (IP: Mule, IB: HA). **i** Immunoprecipitation assay showing an increase in K48-linked ubiquitination of Mule protein (IP: Mule; IB: Lys48-Ub). Immunoblots and IF stainings representative of two or more independent experiments.

UBE2T was found to regulate the protein expression of BRCA1 and p53 via ubiquitination^15,16^. Based on the findings of these studies, UBE2T exerts its physiological and functional roles through protein–protein interaction. To elucidate the underlying mechanism by which UBE2T regulates liver CSCs, we employed tandem affinity purification coupled with mass spectrometry (TAP-MS) through the establishment of SFB-UBE2T stable clones (Supplementary Fig. S4). TAP-MS revealed a list of potential binding targets of UBE2T in the FA pathway, including FANCA, FANCB, FANCG, and FANCL (Supplementary Fig. S5). We focused on potential binding targets of E3 ligase, of which Mcl-1 ubiquitin ligase E3 (Mule; also known as HUWE1, ARF-BP1 or HectH9) showed the highest ranking. Mule is an enormous protein with a size of ~480 kDa and 3 unique peptide sequences that have been recognized by mass spectrometry analysis (Supplementary Fig. S5). We first confirmed the interaction between UBE2T and Mule by reciprocal immunoprecipitation using both tagged and endogenous proteins in HEK293T and HCC cells (Supplementary Fig. S6, Fig. 5a, b). Interestingly, we found that UBE2T not only binds to Mule but also directly regulates Mule expression by ubiquitination. By Western blot analysis, UBE2T expression was found to be inversely correlated with Mule upon overexpression and suppression of UBE2T (Fig. 5c). Treatment with the proteasome inhibitor MG132 restored the expression of Mule in UBE2T-overexpressing Huh7 cells, indicating that UBE2T controls the protein level of Mule by proteasomal degradation (Fig. 5d, e). Further co-localization between UBE2T and Mule was observed with the presence of MG132 (Supplementary Fig. S7). To further demonstrate whether UBE2T mediates the degradation of Mule by ubiquitination, a vector harboring UBE2T with defective E2 activity (UBE2T C86A mutant) was constructed by substituting cysteine at the 86th active site with alanine by site-directed mutagenesis. Impaired ubiquitination of Myc-DDK-tagged UBE2T was observed in the C86A mutant compared to the wild-type UBE2T due to diminished E2 activity (Fig. 5f). Western blot analysis showed that the C86A mutation significantly reversed the suppressive effect of UBE2T overexpression on Mule in Huh7 cells (Fig. 5f). This effect was further confirmed by IF staining showing restoration of cytoplasmic Mule expression in the UBE2T C86A mutant compared to its wild-type form (Fig. 5g). Furthermore, the regulatory role of UBE2T on Mule via ubiquitination was examined by ubiquitination assay. Compared to vector control cells, UBE2T-overexpressing Huh7 cells showed an increase in ubiquitinated Mule, while its effect was abolished when E2 activity was impaired (Fig. 5h). Finally, we found that UBE2T regulated Mule degradation specifically through K48-linked ubiquitination (Fig. 5i).

### UBE2T upregulates β-catenin expression through degradation of Mule

**Fig. 6:**
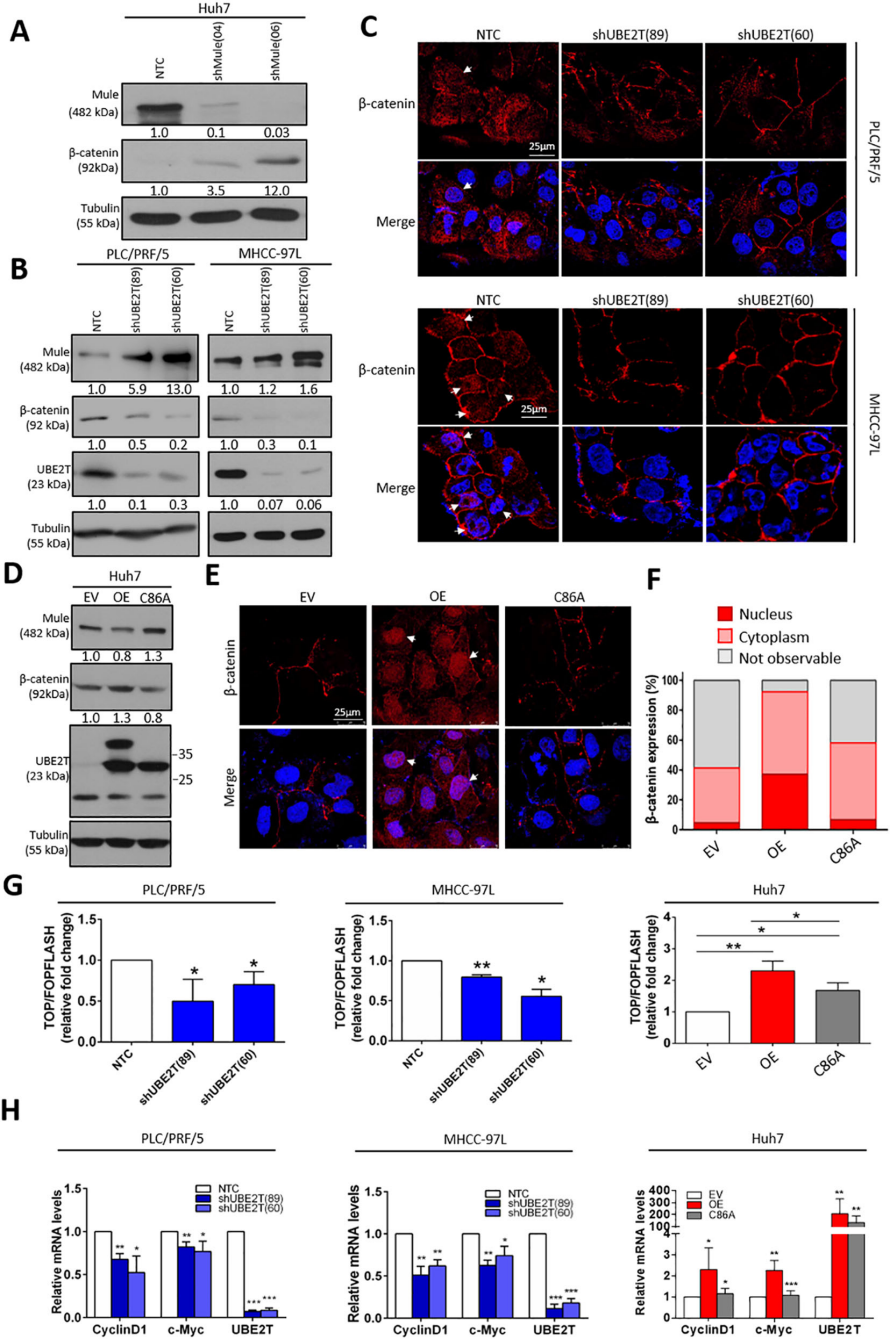
β-catenin as a downstream effect of the UBE2T/Mule complex. **a** Knockdown of Mule led to upregulation of β-catenin expression in Huh7 cells. **b** Knockdown of UBE2T in PLC/PRF/5 and MHCC-97L cells resulted in upregulation of Mule and suppression of β-catenin. **c** IF staining of β-catenin showed a reduction in the total expression and nuclear expression of β-catenin in UBE2T knockdown HCC cells (scale bar: 25 μm). Arrows indicate nuclear expression of β-catenin. **d** Overexpression of UBE2T enhanced β-catenin expression, possibly via degradation of Mule. C86A-transfected HCC cells showed attenuated effects on Mule-mediated β-catenin degradation. **e**, **f** IF staining showed marked upregulation of β-catenin in the nucleus and cytoplasm in OE Huh7 transfectant cells but not C86A mutant cells. Arrows indicate nuclear expression of β-catenin. β-Catenin expression in the nucleus and cytoplasm of Huh7 cells derived from EV, OE, and C86A was quantified. **g** By TOP/FOP assay, transactivating activity of β-catenin was examined in UBE2T-overexpressing and knockdown HCC cells (NTC vs. shUBE2T; EV vs. OE/C86A; OE vs. C86A) (*p < 0.05 and **p < 0.01, *t* test). **h** By qPCR analysis, the expression of β-catenin downstream genes, including Cyclin D1 and c-Myc, was examined in UBE2T-overexpressing and knockdown HCC cells (NTC vs. shUBE2T; EV vs. OE/C86A; OE vs. C86A) (*p < 0.05, **p < 0.01, and ***p < 0.001, *t* test). Immunoblots and IF stainings representative of two or more independent experiments. Immunoblots, IF staining, qPCR, and TOP/FOP assay represent means ± SD in three or more independent experiments (*n* = 3–4).

Mule was previously reported to degrade β-catenin under conditions of hyperactive Wnt signaling in colorectal cancer^17^. Based on this finding, we hypothesized that UBE2T regulates β-catenin expression through facilitating degradation of Mule in HCC cells. To test this hypothesis, we first examined the effect of Mule suppression on the expression of β-catenin in Huh7 cells. ShMule Huh7 cells showed an increase in β-catenin expression compared with control cells (Fig. 6a). Next, we further explored the effect of UBE2T on β-catenin expression. Knockdown of UBE2T was found to stabilize Mule, which led to a concomitant decrease in β-catenin expression in both PLC/PRF/5 and MHCC-97L cells (Fig. 6b). The decrease in β-catenin expression is not obvious in MHCC-97L cells with double knockdown of UBE2T and Mule, which further indicates the role of UBE2T on β-catenin expression via Mule regulation (Supplementary Fig. S8). IF staining revealed less cytoplasmic and nuclear β-catenin protein expression in shUBE2T PLC/PRF/5 and MHCC-97L cells than in control cells (Fig. 6c). Western blot analysis showed that the expression of β-catenin protein was increased in UBE2T OE Huh7 cells, and this effect was abolished in UBE2T C86A-expressing counterparts (Fig. 6d). This result was further confirmed by IF staining, which showed that more cytoplasmic and nuclear β-catenin protein was observed in UBE2T OE cells than in UBE2T C86A cells (Fig. 6e, f). Apart from the protein levels, we explored the effect of UBE2T on the activity of β-catenin in HCC cells. The TOP/FOP reporter assay showed that the transactivation ability of β-catenin was suppressed when UBE2T was repressed, while its activity was elevated in UBE2T-overexpressing cells (Fig. 6g). Reciprocal changes with reduced transactivation ability of β-catenin were observed in the UBE2T C86A mutant. Consistent with the transactivating activity of β-catenin, the expression of known downstream targets of the Wnt/β-catenin pathway, including Cyclin D1 and c-Myc, showed a similar trend in response to alterations in UBE2T expression (Fig. 6h).

### β-catenin is the downstream effector of UBE2T that mediates liver CSC functions

**Fig. 7:**
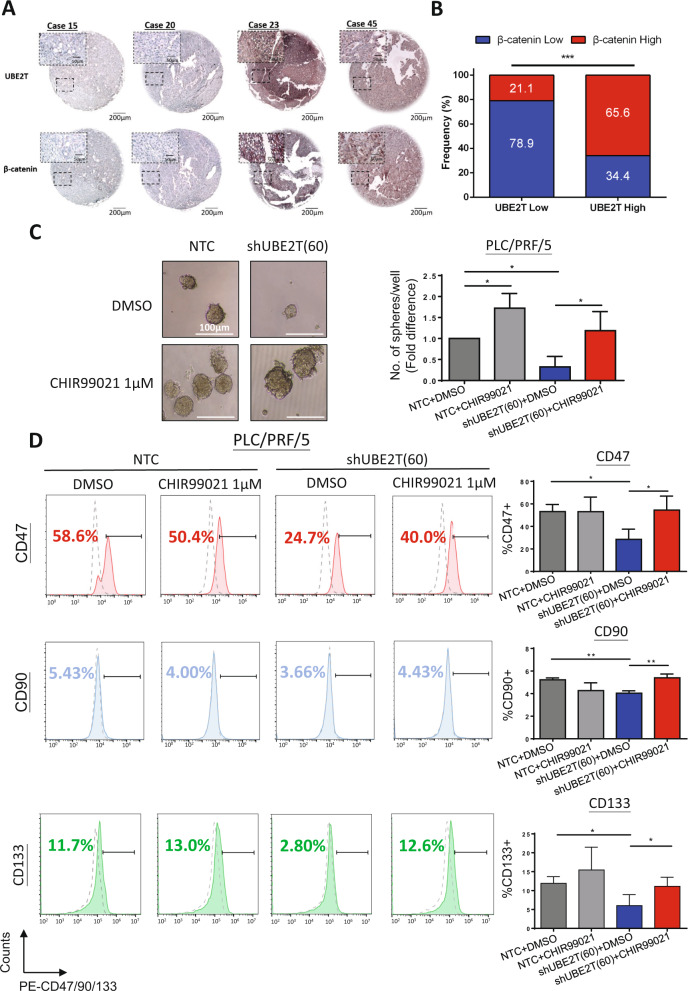
UBE2T regulated liver CSCs through the Wnt/β-catenin signaling cascade. **a** A tissue microarray consisting of 51 tumor tissues and corresponding nontumor liver tissues was subjected to IHC analysis. Cases #23 and #45 showed high expression of both UBE2T and β-catenin, while cases #15 and #20 showed low expression of these proteins. **b** UBE2T expression was significantly correlated with β-catenin expression in a cohort of 51 HCC clinical samples (***p < 0.001, Fisher’s exact test). **c** The addition of CHIR99021 at a dose of 1 μM abolished the inhibitory effect of UBE2T knockdown on the size and number of spheres formed from PLC/PRF/5 cells (*p < 0.05, *t* test). **d** The addition of CHIR99021 at a dose of 1 μM increased the expression of CD47, CD90, and CD133 upon UBE2T knockdown in PLC/PRF/5 cells (*p < 0.05 and **p < 0.01, *t* test). FACS and sphere formation assay represent means ± SD in at least three independent experiments (*n* = 3–4).

To confirm the regulatory role of UBE2T on β-catenin in vivo, the role of UBE2T in regulating β-catenin expression in human HCC clinical samples was explored by correlating UBE2T and β-catenin protein expression by IHC staining. The correlation between UBE2T and β-catenin signaling was further reinforced by the positive correlation between UBE2T and β-catenin expression in a cohort of 51 HCC samples (*p* < 0. 001, Fisher’s exact test) (Fig. 7a, b). To further confirm the role of β-catenin as the downstream effector by which β-catenin mediates CSC functions, we treated UBE2T knockdown HCC cells with CHIR99021, a GSK3β inhibitor, at a dose of 1 μM to investigate whether the effects of UBE2T suppression were eliminated by the activation of β-catenin signaling. We compared the sphere-forming ability and CSC marker expression of shUBE2T HCC cells with or without CHIR99021. We found that the addition of CHIR99021 restored the inhibitory effects of UBE2T knockdown on sphere formation by an average of 2.3-fold (Fig. 7c). Consistently, CHIR99021 restored the expression of liver CSC markers in shUBE2T cells (Fig. 7d). This result demonstrated that UBE2T may regulate liver CSC function, at least in part through the β-catenin signaling cascade.

## Discussion

In an attempt to identify critical molecules/pathways involved in the maintenance and promotion of liver CSCs, we employed an integrative analysis of three publicly available datasets involving HCC clinical and stemness-related data to identify genes that play crucial roles in HCC via the regulation of liver CSCs. Upon analysis, we found enrichment of the interstrand cross-link repair pathway, in which UBE2T was the most upregulated. This result echoed the previous finding that UBE2T was first identified in normal hematopoietic stem cells^10^. UBE2T is the E2 ubiquitin-conjugating enzyme of the FA DNA repair pathway, which is reported to participate in the mitomycin-C-induced DNA repair pathway and activate monoubiquitination of FANCD2, which is essential for the activation of the FA core pathway^18,19^. Consistently, we found that UBE2T was regulated in HCC at both the mRNA and protein levels and was correlated with poor survival in patients. In concordance with our finding, previous studies on UBE2T reported upregulation of UBE2T in many cancer types, such as liver, prostate, lung, nasopharyngeal, and gastric cancer^12,16,20–22^, and UBE2T overexpression has been demonstrated to be related to cell proliferation, migration and invasion, and drug resistance^11,20,23^.

By analyzing the publicly available dataset, we consistently found that UBE2T was upregulated in enriched liver CSCs marked by EPCAM. By using overexpression and knockdown approaches, we found that UBE2T regulates liver CSCs, as evidenced by its effect on self-renewal ability and liver CSC marker expression. Furthermore, we demonstrated that UBE2T regulates CSC frequency and in vivo tumorigenicity in an in vivo limiting dilution assay. This result is consistent with other studies showing the effect of UBE2T on HCC cell growth, probably via regulation of cell cycle regulatory genes, such as p53 and CDK1^16,24^. In addition, we revealed that UBE2T also promoted in vitro invasiveness and induced metastasis in a spontaneous metastasis model. We also demonstrated the putative role of UBE2T in regulating the drug sensitivity of HCC cells to doxorubicin. Taken together, our data strongly support the crucial role of UBE2T in maintaining stem cell-like features in HCC.

Although an increasing number of reports have shown UBE2T overexpression and its oncogenic functions in various malignancies, the underlying mechanism remains unclear. UBE2T has been found to be associated with the Akt signaling cascade^12,25^. However, the exact mechanism of how UBE2T regulates Akt signaling has yet to be elucidated. Given the physiological role of the E2 enzyme, UBE2T was found to promote ubiquitination of target proteins, including FANCD1^18^, BRCA1^15^, and p53^16^. To understand the molecular mechanism of how UBE2T regulates CSC properties and tumor behavior, it is crucial to identify its direct interacting partner. Based on TAP-MS analysis, we identified Mule as one of the two E3 ubiquitin ligases that interacts with UBE2T among a list of potential interacting partners. Consistently, we found that BARD1 was one of the potential candidate partners of UBE2T^15^. However, we did not observe changes in BRCA1 expression upon alteration of UBE2T levels. Mule has been reported as a tumor suppressor in HCC that degrades oncogenic PREX2 proteins via ubiquitination^26^. In addition, Mule expression is suppressed in obesity-induced HCC^27^. We confirmed the role of Mule as a tumor suppressor, as there was a reciprocal correlation between UBE2T and Mule expression in HCC cells upon overexpression and repression of UBE2T. Furthermore, we demonstrated that UBE2T suppressed the expression of Mule in HCC cells by facilitating degradation of Mule via ubiquitination, and this effect was dependent on its E2 enzymatic activity at the conserved cysteine residue C86. Based on this finding, UBE2T may ubiquitinate Mule directly instead of acting as a mediator to transfer ubiquitin to E3 ligases. The direct action of E2 enzymes in the regulation of substrates via ubiquitination is not impossible. For example, UBE2O was found to regulate the direct degradation of AMPK and BMAL1^28,29^. This result may suggest a new noncanonical role of UBE2T in cancer. However, the exact interaction between UBE2T and Mule will be further investigated in the future. E2-E3 interactions are important because the interacting E2s dictate the specific type of inter-Ub linkage either at K48 or K63 and thereby determine the fate of the substrate^30^. We found that UBE2T specifically regulated Mule expression via ubiquitin linkage at K48 and induced ubiquitin-mediated degradation. However, based on our existing data, we cannot exclude the possibility that UBE2T binds to Mule and promote Mule autoubiquitination and degradation. Further investigation is needed to understand how Mule is degraded via interacting with UBE2T.

Mule was previously reported to regulate Wnt signaling in colorectal cancer via degradation of β-catenin and EPHB3^17,31^. In this study, we focused on how Mule regulates β-catenin expression, as the role of β-catenin in the regulation of stemness in HCC has been well reported^32,33^. We found that β-catenin expression was increased in Mule-repressed HCC cells compared to control cells. Furthermore, we found that there is a positive correlation between UBE2T and β-catenin in UBE2T-altered HCC cells and that this relationship was abolished when the E2 activity of UBE2T was impaired. The effect of UBE2T on the activation of the Wnt/β-catenin signaling pathway was further confirmed by the localization and activation of β-catenin transcription. Furthermore, we found that GSK3β inhibitor restored the inhibitory effects of UBE2T knockdown on sphere formation and CSC markers expression. This study suggests that β-catenin is an important downstream effector of UBE2T-mediated CSC functions. However, we could not demonstrate that the degradation of β-catenin is mule-dependent using this approach. In addition, we also observed that the effect of CHIR99021 on spheroid growth is more obvious than the expression of liver CSC markers. The discrepancy may be due to the difference in the time points we assayed for spheroid growth and expression of liver CSC markers. These in vitro results were further confirmed in a cohort of HCC clinical samples that showed a positive correlation between UBE2T and β-catenin. This result is consistent with the correlation between UBE2T and β-catenin in nasopharyngeal carcinoma^12^. Notably, our study has provided more direct mechanistic insight into how UBE2T regulates β-catenin via the degradation of Mule. Finally, we showed that UBE2T may regulate liver CSC function through the regulation of the Wnt/β-catenin signaling cascade.

In conclusion, we demonstrated that UBE2T was upregulated in both liver CSCs and HCCs compared to control cells. The interplay of UBE2T and Mule was found to be critically involved in the regulation of the Wnt/β-catenin signaling cascade (Supplementary Fig. S9). Targeting the UBE2T/Mule/β-catenin signaling cascade may be a new therapeutic strategy for the treatment of HCC patients.

## Supplementary information

Supplementary Methods

Supplementary Figure Legends

Supplementary Fig. S1

Supplementary Fig. S2

Supplementary Fig. S3

Supplementary Fig. S4

Supplementary Fig. S5

Supplementary Fig. S6

Supplementary Fig. S7

Supplementary Fig. S8

Supplementary Fig. S9

Supplementary Table S1

Supplementary Table S2

Supplementary Table S3

Supplementary Table S4

Supplementary Table S5
