# Supplementary material for: The interplay of UBE2T and Mule in regulating Wnt/β-catenin activation to promote hepatocellular carcinoma progression
Source: Cell Death Dis. 2021 Feb 1;12(2):148. doi: 10.1038/s41419-021-03403-6 (PMC7862307; doi:10.1038/s41419-021-03403-6)
Supplement: Supplementary file 1 — Supplementary Methods [file 41419_2021_3403_MOESM1_ESM.docx]

**Supplementary Methods**

**Clinical Characteristics of HCC patients in different cohorts**

For the correlation between UBE2T with clinical pathological features, liver cancer specimens were collected at the time of surgical resection with informed consent from patients at Queen Mary Hospital, Hong Kong, from 1991 to 2013. The age of the patients ranged from 24 to 82 years. There were 50 men and 17 women. Serum hepatitis B surface antigen was positive in 61 patients (91%). Tumors were staged according to the pathological tumor-node-metastasis (pTNM) staging system, 1997 version. 28 patients are at stage I to II (early stage) while 39 patients are at stage III to IV (late stage). 50.7% of patients had venous infiltration at the time of diagnosis with tumor size ranged from 2 to 18 cm, with a mean of 8.01cm.

We also analyzed the correlation between UBE2T with survival analysis from the Cancer Genome Atlas Liver Hepatocellular Carcinoma (TCGA-LIHC) dataset. The age of the patients ranged from 16 to 90 years. There were 255 men and 122 women. Serum hepatitis B surface antigen was positive in 107 patients (28.8%). Tumors were staged according to the Neoplasm Disease Stage American Joint Committee on Cancer Code. 262 patients are at stage I to II (early stage) while 91 patients are at stage III to IV (late stage). 111 (30%) of patients had venous infiltration at the time of diagnosis with tumor initial weight ranged from 10 to 2190g, with a mean of 273g (median:160). There are totally 30 HCC patients with prior sorafenib treatment. The age of the patients ranged from 23 to 85 years. There were 14 men and 16 women. Serum hepatitis B surface antigen was positive in 1 patient. Tumors were staged according to the Neoplasm Disease Stage American Joint Committee on Cancer Code. 16 patients are at stage I to II (early stage) while 13 patients are at stage III to IV (late stage). 13(43.3%) of patients had venous infiltration at the time of diagnosis with tumor initial weight ranged from 20 to 1580g, with a mean of 281g (median:220).

In addition, two publicly available datasets including GSE5975 and GSE25097 from Chinese origin were employed for data analysis in which majority of the patients are HBV-positive.

**Plasmids**

Plasmid pCMV6-Myc-DDK-UBE2T was purchased from OriGene (Rockville, MD, USA). DDK is the same as FLAG®. pCMV6-Myc-DDK-UBE2T C86A mutant was constructed by site-directed mutagenesis from GenScript (Piscataway, NJ, USA). pMH-SFB vector was provided by Dr. Michael Huen (The University of Hong Kong, China). pcDNA3.1-HA-ubiquitin was kindly provided by Dr. Judy Yam (The University of Hong Kong, China).

**Chemicals**

Doxorubicin was purchased from EBEWE Pharma. CHIR99021 (SML 1046) was purchased from Sigma.

**Tissue microarray**

Archived paraffin-embedded pathological specimens from primary HCC patients were collected along with complete clinical and pathological data at the Sun Yat Sen University Cancer Center. All samples were anonymous. This study was approved by the Institute Research Medical Ethics Committee. None of the patients had received radiotherapy or chemotherapy before surgery.

**Lentiviral-based transfection into HCC cells**

For suppression of UBE2T and Mule in HCC cells, shRNA sequences against human UBE2T and Mule were cloned to pLKO.1 vector (Addgene). Transduced cells were selected with 2 µg/mL puromycin. **Supplementary Table S4** lists the sequences of the UBE2T, mule and NTC used. For overexpression, Stable HCC cells ectopically overexpressing pCMV6-Myc-DDK-UBE2T and pCMV6-Myc-DDK-UBE2T C86A were selected by G418 at mg/mL (GoldBio) treatment for 2 weeks.

**Sphere formation assay**

HCC cells were plated onto 24-well plate pre-coated with 1% polyHEMA (MilliporeSigma). Cells were cultured in DMEM/F12 medium (Gibco, Invitrogen) supplemented with 4 µg/mL insulin (MilliporeSigma) and B27 (Gibco, Invitrogen) for 10 days for PLC/PRF/5. 20 ng/mL EGF (Sigma-Aldrich) and 20 ng/mL basic FGF (Invitrogen) were also applied to the sphere cultures of MHCC-97L and Huh7 cells.

**Annexin V apoptosis assay**

Cells were stained by FITC-conjugated Annexin V (BioVision) and PI (Invitrogen) in Annexin V binding buffer (BD Biosciences) at room temperature for 15 mins. Apoptosis percentage was determined using BD Accuri C6 flow cytometer and FACSDiva software (BD Biosciences).

**Ubiquitination assay**

pcDNA3.1-HA-ubiquitin was co-transfected with pCMV6-Entry empty vector, pCMV6-Myc-DDK-UBE2T or its C86A mutant construct for 24 hrs and then treated with 20 µM MG132 for 6 hrs. The cells were lysed with RIPA buffer (20mM Tris-HCl (pH 7.5), 150mM NaCl, 1mM Na_2_EDTA, 1mM EGTA, 1% NP40, 0.025%SDS, 1% sodium deoxycholate, 2.5mM sodium pyrophosphate, 1mM β-glycerophosphate, 1mM Na_3_VO_4_, 1µg/mL leupeptin)) supplemented with protease inhibitor cocktail (Roche) and phosphatase inhibitors (Roche).The protein lysate was immunoprecipitated overnight with anti-Mule (ab70161, Abcam) antibody at 4 °C with gentle rotation, later with Protein A agarose (Cell Signaling Technology) for 6 hrs at 4 °C with gentle agitation. Immunoprecipitates were washed three times with 1% TritonX-100-PBS, eluted by boiling in 2X SDS loading buffer for 10 mins and subjected to immunoblotting analyses. Primary antibodies against HA (1:1000, 66006-1-Ig, Proteintech), ubiquitin (Lys48-specific) (1:1000, 05-1307, Millipore) were used for detection of ubiquitinated Mule.

**Migration and invasion assays**

***.*** The migration assay was performed as described. The cell invasion assay was performed with self-coated Matrigel (BD Biosciences) on the upper surface of a transwell chamber. The cells that had invaded through the extracellular matrix layer to the lower membrane surface were fixed with 2% PFA in PBS and stained with crystal violet. Photographs of four randomly selected fields of the fixed cells were captured and the cells were counted.

**Flow cytometric analysis**

Cells were stained by PE-conjugated CD47, CD90 (BD Biosciences) and CD133 (Miltenyi Biotec) antibodies in PBS with 2% FBS at 4°C for 30-60 mins. Isotype-matched mouse immunoglobulins served as controls. Samples were analyzed using BD Accuri C6 flow cytometer and FACSDiva software (BD Biosciences).

**Immunoprecipitation**

HCC cells were transfected with pCMV6-Entry empty vector, pCMV6-Myc-DDK-UBE2T or its C86A mutant construct by Lipofectamine 2000. The transfected cells were lysed by NETN buffer (20mM Tris-HCl (pH 8), 150mM NaCl, 1mM EDTA, 0.5% NP-40) with addition of protease inhibitor cocktail (Roche) and phosphatase inhibitors (Roche). Concentration of the protein lysate was measured by Bio-rad protein assay. The protein lysate was incubated with anti-UBE2T (#10105-2-AP, Proteintech) antibody or anti-Mule (ab70161, Abcam) antibody together with Protein A agarose (Cell Signaling Technology) at 4^o^C overnight with gentle agitation. Normal rabbit IgG served as control. The immunoprecipitates were eluted by boiling in 2X SDS loading buffer for 5 mins and then subjected to SDS-PAGE and immunoblotting analysis.

**Luciferase reporter assay**

β-catenin activity was examined using luciferase reporter assay of TCF/LEF-dependent transcription (TOP/FOPFLASH reporter assay). Either Firefly luciferase pSuper8XTOPflash or pSuper8XFOPflash constructs (gifts from Dr. Moon R, University of Washington, USA), together with Renilla luciferase construct pRL-SV40 (Promega, Madison, WI, USA) for normalization of transfection efficiency, were transfected using Lipofectamine® 2000. Luciferase activities were assayed using Dual-Luciferase® Reporter Assay System (Promega) according to manufacturer’s protocol.

**RNA extraction and quantitative PCR (qRT-PCR) analysis**

Total RNA was isolated using TRIzol reagent according to the manufacturer’s protocol (Invitrogen). Complementary DNA (cDNA) was synthesized using PrimeScript RT Reagent Kit (Takara) according to the manufacturer’s instructions and then subjected to qPCR with BrightGreen 2x qPCR Master mix (Applied Biological Materials) using QuantStudio 7 Flex Read Time PCR System (Applied Biosystems) with primers specific to the sequences of genes of interest which were provided in **Supplementary Table S5**. Relative expression differences were calculated using 2^−ΔΔCT^method with reference to β-actin/GAPDH.

**Western blot analysis**

Whole cell lysates were extracted using RIPA buffer supplemented with protease inhibitor cocktail. Protein lysate were separated by SDS-polyacrylamide gel electrophoresis (SDS-PAGE) and transferred to polyvinylidene difluoride membrane (Millipore) for western blot analyses. Primary antibodies against UBE2T (1:1000, #10105-2-AP, Proteintech), Mule (1:1000, ab70161, Abcam), β-catenin (1:1000, 8490S, Cell Signaling Technology) and α-tubulin (1:5000, T9026, Sigma-Aldrich) were incubated at 4°C overnight. After washing, the membrane was incubated with horseradish peroxidase-conjugated anti-mouse or rabbit antibody (GE HealthCare). The signals were visualized using the enhanced chemiluminescence method.

**Immunofluorescence staining**

For staining the spheroids, cells grown on glass coverslips were fixed in 4% PFA, permeabilized with 0.1% Triton X-100 and stained with anti-DDK (1:100, TA50011-100, Origene), anti-Mule (1:100, ab70161, Abcam) and anti-β-catenin (1:100, #610153, BD Biosciences) antibodies. The cells were then washed with PBS, incubated with FITC- conjugated and/or TR-conjugated secondary antibodies (Life Technologies) wherever appropriate, and counterstained with DAPI. Fluorescence signal was then visualized using Leica TCS SPE confocal microscope.

**Immunohistochemistry**

For paraffin-embedded HCC tissues, sections were deparaffinized in xylene and rehydrated in graded alcohols and distilled water. Slides were processed for antigen retrieval by a standard microwave heating technique in Tris-EDTA buffer. Endogenous peroxidase activities were quenched using 3% hydrogen peroxide. The sections were immersed in serum free-protein block solution (Dako). Specimens were subsequently incubated with anti-UBE2T (1:200, #10105-2-AP, Proteintech) and anti-β-catenin (1:200, #610153, BD Biosciences) antibodies. The sections were then washed thoroughly and incubated with anti-mouse or rabbit Envision^TM^ HRP-conjugated secondary antibody (Dako). Positive signals were visualized using Liquid DAB+ Substrate-Chromogen System (DAKO). Sections were counterstained with Mayer’s hematoxylin followed by examination using light microscope. For quantitation of UBE2T and β-catenin expression, the stained sections were assessed with no prior knowledge of the clinico-pathological data for the patients. Each specimen was individually scored from 1 to 4 in terms of percentage (P) of expression, ≤10% stained positive, ≤25% stained positive, <50% stained positive and ≥50 stained positive, respectively. For intensity (I), each specimen was individually score from 1 to 3, 1 represents weak; 2 represents moderate; and 3 represents strong. Quick score was obtained by multiplying the percentage of positive cells (P) by the intensity (I). Formula: Q = P x I; Maximum = 12. The specimens with score <6 belong to the “low expression” group while those ≥6 belong to “high expression”.

**Clinico-pathological correlation analysis and survival analysis**

Clinico-pathological features of human HCC were correlated with mRNA levels of UBE2T in clinical human HCC tissues. Correlations were performed for features including gender, age, number of tumor nodules, tumor size, tumor encapsulation, hepatitis B surface antigen, AFP level, tumor staging, cellular differentiation, cirrhosis, tumor relapse, and venous invasion using SPSS 20.0 software. Analysis and grading of pathological-tumor-nodes-metastasis tumor staging was performed by a pathologist. Survival analysis was determined using the Kaplan-Meier method followed by log-rank test.
