# Supplementary material for: The interplay of UBE2T and Mule in regulating Wnt/β-catenin activation to promote hepatocellular carcinoma progression
Source: Cell Death Dis. 2021 Feb 1;12(2):148. doi: 10.1038/s41419-021-03403-6 (PMC7862307; doi:10.1038/s41419-021-03403-6)
Supplement: Supplementary file 2 — Supplementary Figure Legends [file 41419_2021_3403_MOESM2_ESM.docx]

**Ho et al_ Supplementary Figure Legends**

**Supplementary Fig. S1**. **Expression of UBE2T expression in a panel of HCC cell lines.** By Western blot analysis, MIHA, a non-tumorigenic normal liver cell line, showed the lowest expression of UBE2T, when compared with a variety of HCC cell lines. UBE2T was found to be preferentially expressed in HCC cell lines including PLC/PRF/5 and MHCC-97L.

**Supplementary Fig. S2**. **UBE2T regulates the sensitivity of HCC cells to sorafenib and lenvatinib treatments.** Compared to NTC (non target control) cells, shUBE2T cells derived from PLC/PRF/5 and MHCC-97L cells showed more cell death in response to sorafenib and lenvatinib treatments at 10 and 40 µM, respectively, for 48 hrs. Compared to EV (empty vector) control cells, UBE2T OE (overexpressing) cells showed a lower percentage of apoptosis in response to sorafenib and lenvatinib treatments for 48 hrs (**p*<0.05 & ***p*<0.01, *t* test).

**Supplementary Fig. S3**. **UBE2T expression correlated with survival of HCC patients who received sorafenib treatment. (A)** Patients with high UBE2T expression after sorafenib treatment would have shorter overall survival than those with lower UBE2T expression (p=0.0299). **(B)** Among these 30 HCC patients who received prior sorafenib treatments, UBE2T expression was not correlated with TNM stage.

**Supplementary Fig. S4**. **Identification of Mule as the direct binding partner by TAP-MS.** UBE2T was modified to add N-terminal epitope tag (S protein tag-Flag tag-Streptavidin binding peptide) for affinity purification. pMH-SFB vector containing SFB-tagged UBE2T ORF was transfected to HEK293T for overexpression of the modified protein. Upon transfection, single clones of UBE2T transfectants were identified and isolated by limiting dilution and expansion after puromycin selection. To the end, we have isolated 4 clones and verified SFB-UBE2T expression by western blot analysis. Among these four clones, clone #4 showed highest expression of SFB-UBE2T (highlighted in red), and thus the protein lysate derived from clone 4 was extracted for further mass spectrometry analysis.

**Supplementary Fig. S5**. **Mule was identified as the sole E3 ligase upon mass spectrometry analysis**. After mass spectrometry analysis, several genes in Fanconi anemia (FA) pathway including FANCA, FANCB, FANCG and FANCL were pulled down. E3 ligases were screened in the list of potential protein partners of UBE2T because of high affinity of E2 enzyme for E3 ligase and their biological role in ubiquitination. Mule was identified as the sole E3 ligase which may potentially bind to UBE2T in the mass spectrometry results. 3 unique peptide sequences of Mule (circled in red) were identified upon mass spectrometry analysis.

**Supplementary Fig. S6**. **Identification of Mule as the potential binding partner of UBE2T.** Upon mass spectrometry analysis, Mule, the only E3 ligase in the list was identified with peptide hit of 3. Mass spectrometry result was confirmed by immunoprecipitation assay in HEK293T cells which showed physical interaction between Mule and UBE2T (IP: Streptavidin; IB: Mule and UBE2T).

**Supplementary Fig. S7**. **Co-localization between UBE2T and Mule was found upon MG132 treatment.** UBE2T-overexpressing Huh7 cells were subjected to 20 µM MG132 treatment for 6 hrs. Mule expression in UBE2T OE cells was reduced when compared to control cells. MG132 treatment increased the level of Mule, indicating that UBE2T regulates Mule by proteasomal degradation. Co-localization between UBE2T and Mule indicated by white arrows(scale bar: 25 μm).

**Supplementary Fig. S8**. **UBE2T regulates β-catenin expression via regulation of Mule degradation.** Western blot analysis showed the expression of UBE2T, Mule and β-catenin in shUBE2T and shUBE2T/shMule cells derived from MHCC-97L cells.

**Supplementary Fig. S9.** UBE2T regulates liver CSCs through regulation of Mule-mediated β-catenin degradation.
