# Supplementary figures and images for: The interplay of UBE2T and Mule in regulating Wnt/β-catenin activation to promote hepatocellular carcinoma progression
Source: Cell Death Dis. 2021 Feb 1;12(2):148. doi: 10.1038/s41419-021-03403-6 (PMC7862307; doi:10.1038/s41419-021-03403-6)

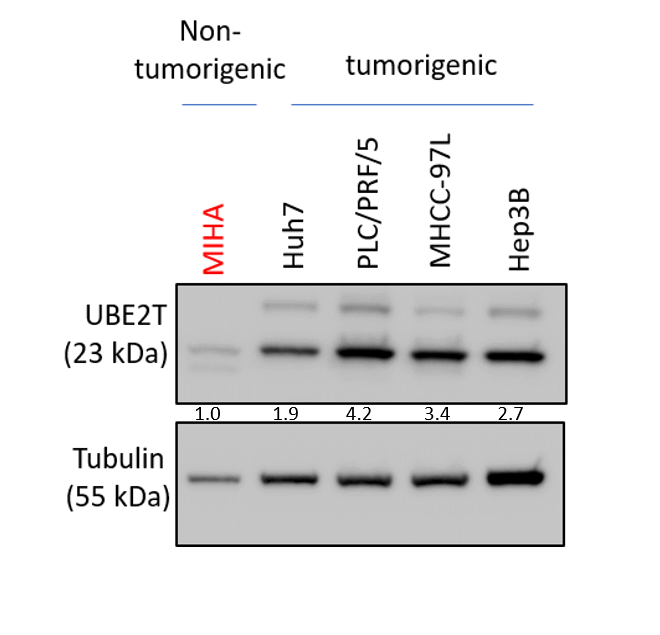

Supplement: Supplementary file 3 — Supplementary Fig. S1 [file 41419_2021_3403_MOESM3_ESM.tif]

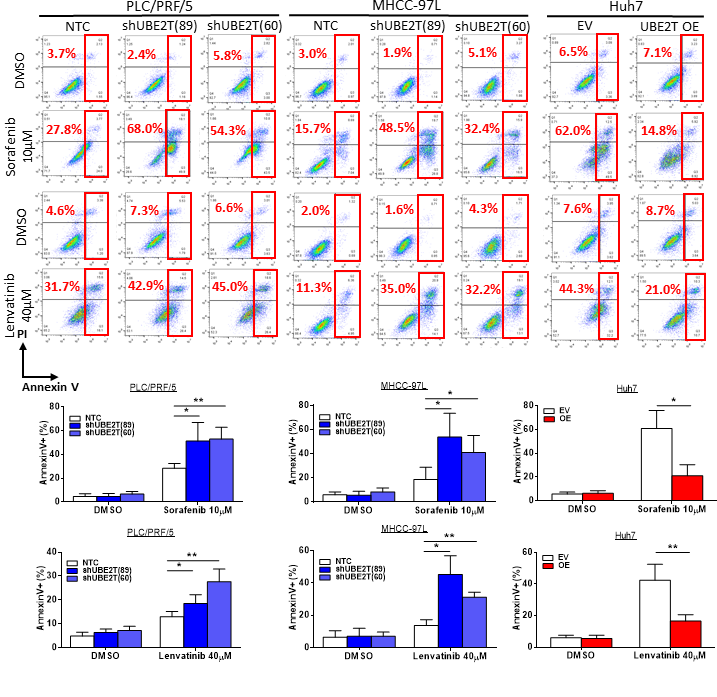

Supplement: Supplementary file 4 — Supplementary Fig. S2 [file 41419_2021_3403_MOESM4_ESM.tif]

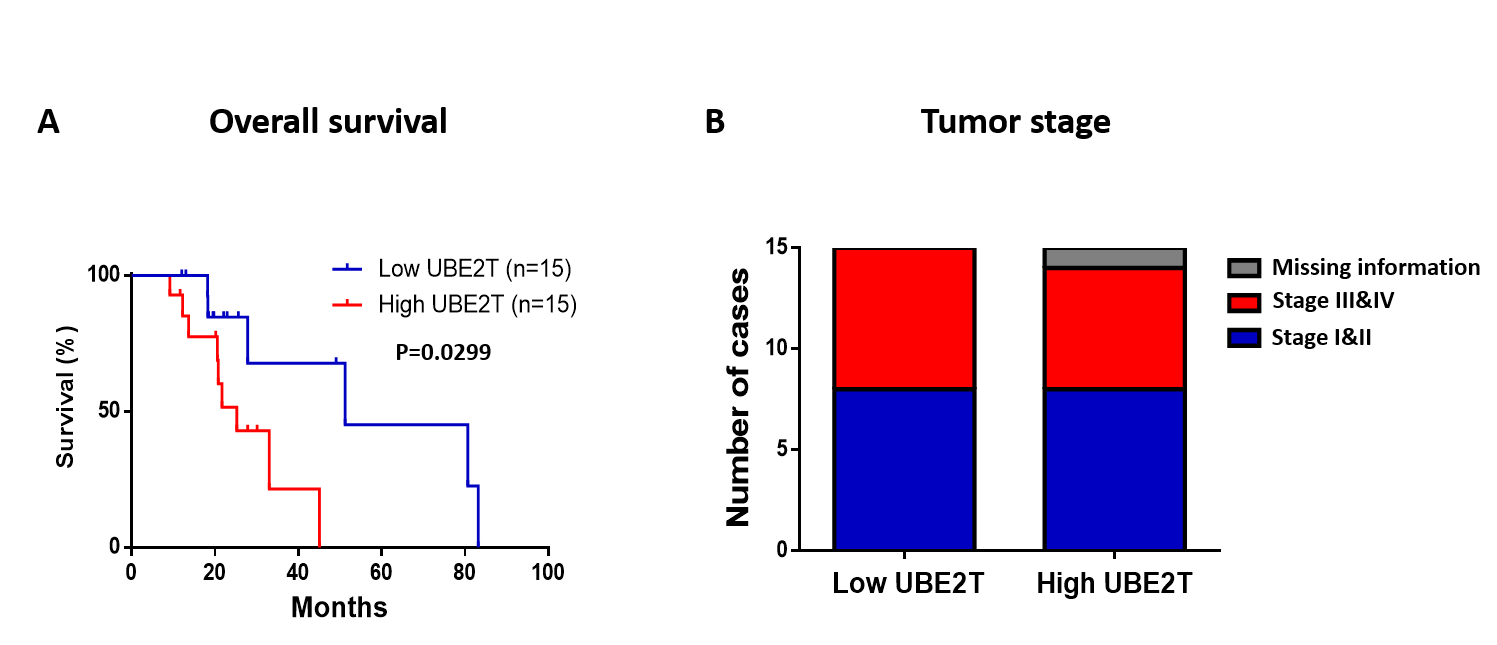

Supplement: Supplementary file 5 — Supplementary Fig. S3 [file 41419_2021_3403_MOESM5_ESM.tif]

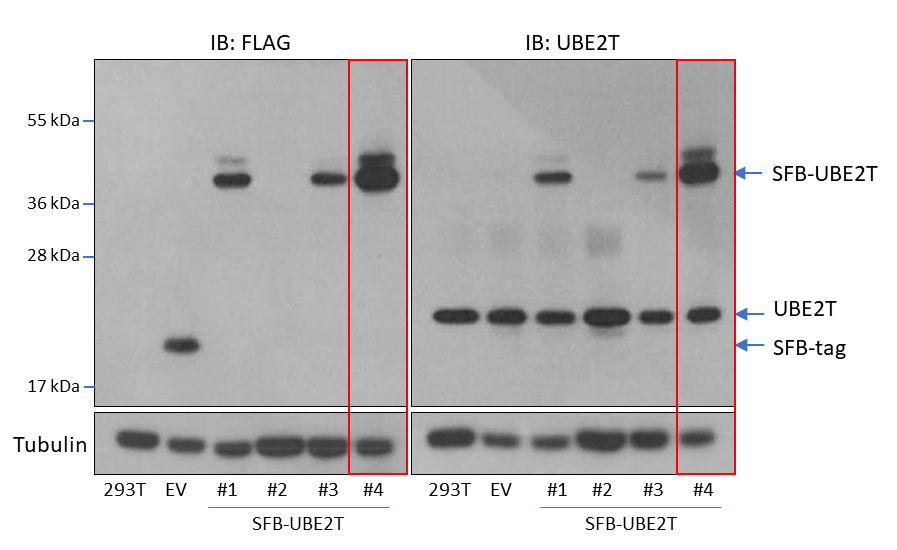

Supplement: Supplementary file 6 — Supplementary Fig. S4 [file 41419_2021_3403_MOESM6_ESM.tif]

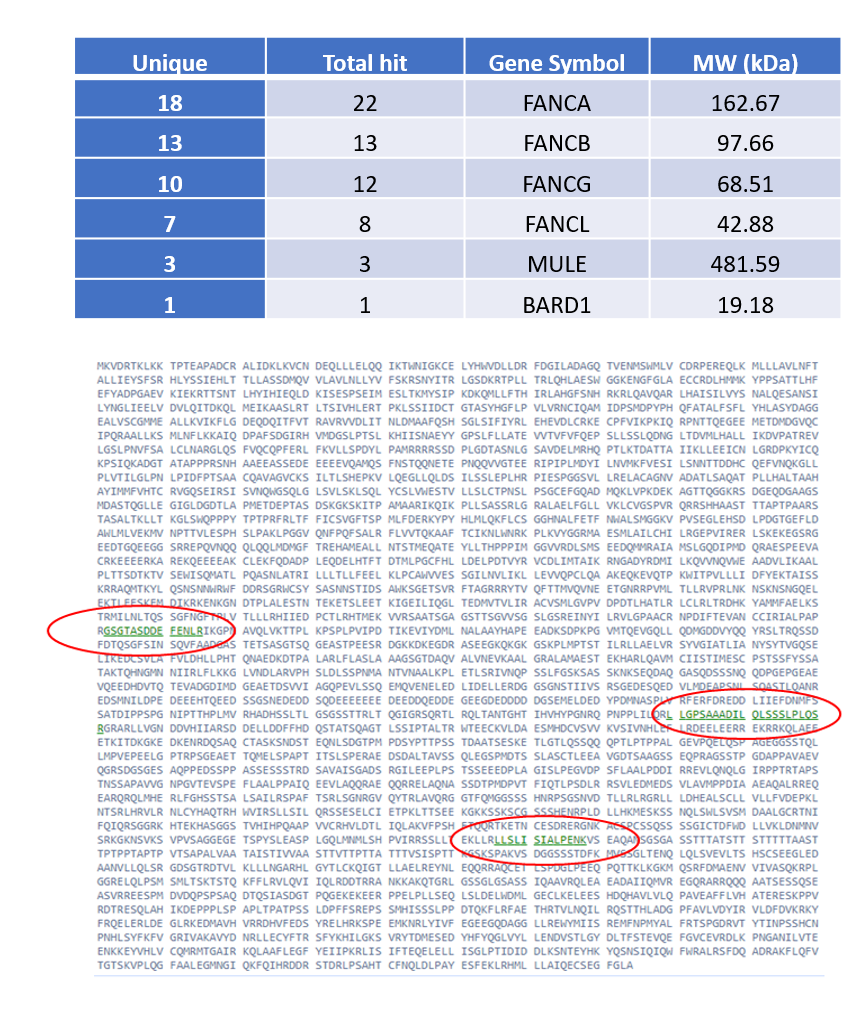

Supplement: Supplementary file 7 — Supplementary Fig. S5 [file 41419_2021_3403_MOESM7_ESM.tif]

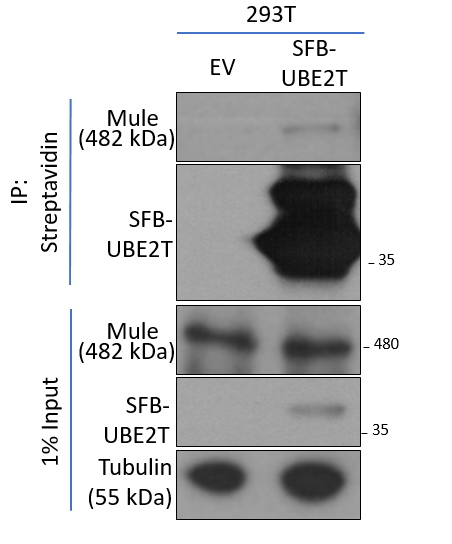

Supplement: Supplementary file 8 — Supplementary Fig. S6 [file 41419_2021_3403_MOESM8_ESM.tif]

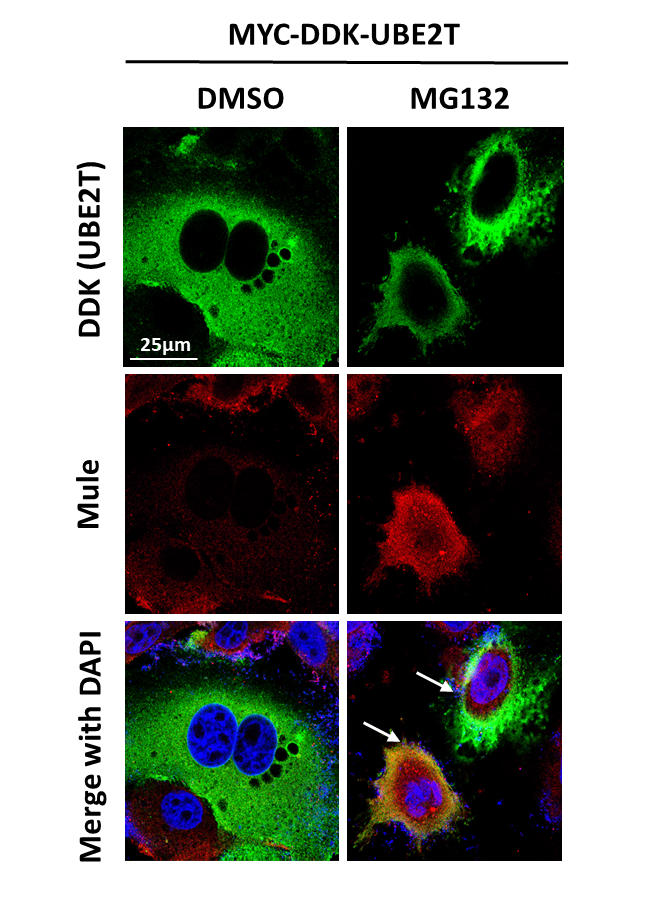

Supplement: Supplementary file 9 — Supplementary Fig. S7 [file 41419_2021_3403_MOESM9_ESM.tif]

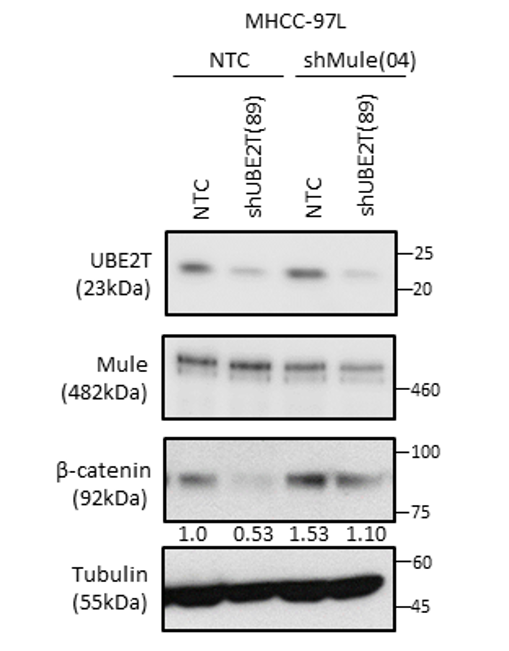

Supplement: Supplementary file 10 — Supplementary Fig. S8 [file 41419_2021_3403_MOESM10_ESM.tif]

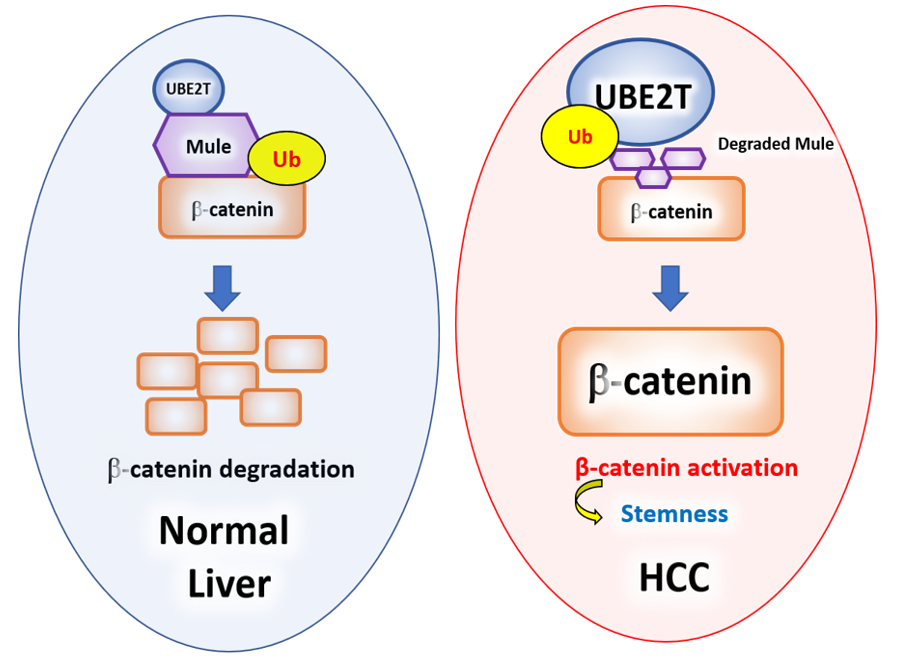

Supplement: Supplementary file 11 — Supplementary Fig. S9 [file 41419_2021_3403_MOESM11_ESM.tif]
