# Supplementary material for: The interplay of UBE2T and Mule in regulating Wnt/β-catenin activation to promote hepatocellular carcinoma progression
Source: Cell Death Dis. 2021 Feb 1;12(2):148. doi: 10.1038/s41419-021-03403-6 (PMC7862307; doi:10.1038/s41419-021-03403-6)
Supplement: Supplementary file 12 — Supplementary Table S1 [file 41419_2021_3403_MOESM12_ESM.docx]

**Supplementary Table S1**. **Top upregulated genes by ranking fold change in each comparison**

| Ranking | Gene |
| --- | --- |
| 1 | AFP |
| 2 | COL2A1 |
| 3 | NTS |
| 4 | PRAME |
| 5 | TRIM71 |
| 6 | DLK1 |
| 7 | SIX2 |
| 8 | CLEC2L |
| 9 | MNX1 |
| 10 | SLC7A10 |
