# Supplementary material for: The interplay of UBE2T and Mule in regulating Wnt/β-catenin activation to promote hepatocellular carcinoma progression
Source: Cell Death Dis. 2021 Feb 1;12(2):148. doi: 10.1038/s41419-021-03403-6 (PMC7862307; doi:10.1038/s41419-021-03403-6)
Supplement: Supplementary file 13 — Supplementary Table S2 [file 41419_2021_3403_MOESM13_ESM.docx]

**Supplementary Table S2**. **Clinico-pathological correlation of UBE2T expression in human HCCs**

| **Clinico-pathological features** | **UBE2T mRNA** | | **p value**  **(Fisher-exact test)** |
| --- | --- | --- | --- |
|  | **T:NT < 8** | **T:NT ≥ 8** |  |
| **Gender** |  |  |  |
| **Male** | 21(75.0%) | 29 (74.4%) | 1.000 |
| **Female** | 7 (25.0%) | 10 (25.6%) |  |
| **Venous invasion** |  |  |  |
| **Absent** | 18 (64.3%) | 15 (38.5%) | 0.049* |
| **Present** | 10 (35.7%) | 24 (61.5%) |  |
| **Tumor encapsulation** |  |  |  |
| **Absent** | 14 (53.8%) | 27 (73.0%) | 0.179 |
| **Present** | 12 (46.2%) | 10 (27.0%) |  |
| **Tumor microsatellite** |  |  |  |
| **Absent** | 14 (53.8%) | 15 (38.5%) | 0.309 |
| **Present** | 12 (46.2%) | 24 (61.5%) |  |
| **Cellular differentiation** |  |  |  |
| **0– 3** | 13 (56.5%) | 12 (32.4%) | 0.105 |
| **4 – 6** | 10 (43.5%) | 25 (67.6%) |  |
| **Tumor size** |  |  |  |
| **≤5 cm** | 11 (47.8%) | 7 (18.9%) | 0.023* |
| **>5 cm** | 12 (52.2%) | 30 (81.1%) |  |
| **Cirrhotic liver** |  |  |  |
| **Normal and chronic hepatitis** | 9 (39.1%) | 24 (64.9%) | 0.065 |
| **cirrhosis** | 14 (60.9%) | 13 (35.1%) |  |
| **Tumor stage** |  |  |  |
| **I/II** | 16 (57.1%) | 12 (30.8%) | 0.045* |
| **III/IV** | 12 (42.9%) | 27 (69.2%) |  |

*P<0.05; N= 67
