# Supplementary material for: The interplay of UBE2T and Mule in regulating Wnt/β-catenin activation to promote hepatocellular carcinoma progression
Source: Cell Death Dis. 2021 Feb 1;12(2):148. doi: 10.1038/s41419-021-03403-6 (PMC7862307; doi:10.1038/s41419-021-03403-6)
Supplement: Supplementary file 14 — Supplementary Table S3 [file 41419_2021_3403_MOESM14_ESM.docx]

**Supplementary Table S3**. Effect of UBE2T suppression on tumorigenicity of PLC/PRF/5 and MHCC-97L cells using lentiviral based knockdown approach, and Huh7 cells with ectopic overexpression. (A) Subcutaneous *in vivo* tumor development in NOD/SCID mice of shUBE2T cells and non-target control cells from PLC/PRF/5. (B) Subcutaneous *in vivo* tumor development in NOD/SCID mice of shUBE2T cells and non-target control cells from MHCC-97L. (C) Subcutaneous *in vivo* tumor development in NOD/SCID mice of UBE2T OE cells and empty vector control cells from Huh7.

A. Primary engraftment of PLC/PRF/5 cells

| PLC/PRF/5 | Tumor incidence rate | | | Extreme limiting dilution | | |
| --- | --- | --- | --- | --- | --- | --- |
|  | 1000 cells | 10000 cells | 50000 cells | Estimated CSC frequency | 95% CI | P-value |
| NTC | 1/8 | 3/8 | 6/8 | 1/27903 | 1/56286-1/13834 |  |
| shUBE2T(89) | 0/8 | 1/8 | 2/8 | 1/143347 | 1/450782-1/45584 | 0.0073** |
| shUBE2T(60) | 0/8 | 0/8 | 2/8 | 1/218045 | 1/863707-1/55047 | 0.0021** |

B. Primary engraftment of MHCC-97L cells

| MHCC-97L | Tumor incidence rate | | | Extreme limiting dilution | | |
| --- | --- | --- | --- | --- | --- | --- |
|  | 500 cells | 1000 cells | 10000 cells | Estimated T-IC frequency | 95% CI | P-value |
| NTC | 8/8 | 7/8 | 8/8 | 1/279 | 1/556-1/140 |  |
| shUBE2T(89) | 4/8 | 5/8 | 5/8 | 1/3825 | 1/7969-1/1836 | <0.0001*** |
| shUBE2T(60) | 3/8 | 3/8 | 6/8 | 1/4032 | 1/8380-1/1940 | <0.0001*** |

C. Primary engraftment of Huh7 cells

| Huh7 | Tumor incidence rate | | | Extreme limiting dilution | | |
| --- | --- | --- | --- | --- | --- | --- |
|  | 1000 cells | 5000 cells | 10000 cells | Estimated T-IC frequency | 95% CI | P-value |
| EV | 0/4 | 1/4 | 1/4 | 1/28065 | 1/113104-1/6964 |  |
| OE | 1/4 | 3/4 | 3/4 | 1/5010 | 1/11509-1/2181 | 0.0195* |
