# Supplementary material for: The interplay of UBE2T and Mule in regulating Wnt/β-catenin activation to promote hepatocellular carcinoma progression
Source: Cell Death Dis. 2021 Feb 1;12(2):148. doi: 10.1038/s41419-021-03403-6 (PMC7862307; doi:10.1038/s41419-021-03403-6)
Supplement: Supplementary file 15 — Supplementary Table S4 [file 41419_2021_3403_MOESM15_ESM.docx]

**Supplementary Table S4. shRNA sequences.**

| shRNA | Sequence (5'-3') |
| --- | --- |
| NTC | CCGGTTGTGCTCTTCATCTTGTTGCCGGCAACAAGATGAAGAGCACCAATTTTTG |
| shUBE2T(89) | CCGGGTCCTGGTTCATCTTAGTTAACTCGAGTTAACTAAGATGAACCAGGACTTTTT |
| shUBE2T(60) | CCGGTGAGGAAGAGATGCTTGATAACTCGAGTTATCAAGCATCTCTTCCTCATTTTTTG |
| shMule(04) | CCGGCCACACTTTCACAGATACTATCTCGAGATAGTATCTGTGAAAGTGTGGTTTTTG |
| shMule(06) | CCGGCGACGAGAACTAGCACAGAATCTCGAGATTCTGTGCTAGTTCTCGTCGTTTTTG |
