# Supplementary material for: The interplay of UBE2T and Mule in regulating Wnt/β-catenin activation to promote hepatocellular carcinoma progression
Source: Cell Death Dis. 2021 Feb 1;12(2):148. doi: 10.1038/s41419-021-03403-6 (PMC7862307; doi:10.1038/s41419-021-03403-6)
Supplement: Supplementary file 16 — Supplementary Table S5 [file 41419_2021_3403_MOESM16_ESM.docx]

**Supplementary Table S5. Primer sequences for qRT-PCR.**

| Genes | Forward primer (5’-3’) | Reverse primer (5’-3’) |
| --- | --- | --- |
| UBE2T | TTAGGTGGAGCCAACACACC | GAGGGATGGTCTCCAAGCAC |
| Mule | AGCGCCTCATTTCCATCTTCA | TGCAGGGGTACCTTGGAAGT |
| Cyclin D1 | GATGCCAACCTCCTCAACGA | GGAAGCGGTCCAGGTAGTTC |
| c-Myc | CGTCCTCGGATTCTCGCTC | GCTGGTGCATTTTCGGTTGT |
| GAPDH | CCTCCTGGCGTCGTGATTAGTG | CAGAGGGCTACAATGTGATGG |
| β-actin | GTGGGGCGCCCCAGGCACCA | CTCCTTAATGTCACGCACGATTTC |
